# Detection of residual consciousness using EEG indicators related to rectal perception: protocol for a diagnostic accuracy study

**DOI:** 10.3389/fneur.2026.1802303

**Published:** 2026-05-20

**Authors:** Yilin Zhao, Yitong Lian, Yinliang Qi, Steven Laureys, Haibo Di, Weiqiao Zhao

**Affiliations:** 1 Zhejiang-Belgium Joint Laboratory for Disorders of Consciousness, Hangzhou Normal Univeristy, Hangzhou, China; 2 School of Basic Medical Sciences, Hangzhou Normal University, Hangzhou, China; 3 General Department of Hyperbaric Oxygen, The Second People's Hospital of Hefei, Hefei Affiliated Hospital of Anhui Medical University, Hefei, Anhui, China; 4 Joint International Research Unit on Consciousness, CERVO Brain Research Centre, Laval University, Québec, QC, Canada; 5 Coma Science Group, GIGA-Consciousness, University of Liège, Liège, Belgium

**Keywords:** diagnosis, disorders of consciousness, gut-brain axes, interoception, rectum

## Abstract

**Introduction:**

Accurate diagnosis of disorders of consciousness (DoC), including unresponsive wakefulness syndrome (UWS) and minimally conscious state (MCS), remains a long-standing and unresolved challenge in clinical practice. Current diagnostic frameworks rely predominantly on behavioral responsiveness, with the Coma Recovery Scale–Revised (CRS-R) serving as the clinical gold standard. However, behavior-based evaluation is intrinsically vulnerable to examiner subjectivity and cognitive–motor dissociation, contributing to misdiagnosis rates of up to 40%. From a theoretical perspective, consciousness comprises both external awareness and self-awareness, yet existing DoC assessments focus almost exclusively on external sensory processing, leaving self-awareness substantially underassessed. This imbalance highlights a critical gap that motivates for complementary assessment approaches targeting underexplored dimensions of consciousness.

**Methods and analysis:**

This study protocol aims to develop an EEG-based paradigm and evaluate its feasibility for assessing gut-related interoceptive processing in patients with DoC, using controlled, non-invasive rectal balloon distension. Standardized stimulation procedures, synchronized EEG acquisition, and predefined analytical pipelines will be implemented to characterize the temporal and spatial features interoceptive event-related potentials.

**Results:**

Electrophysiological and statistical analyses will be conducted to assess the feasibility, signal characteristics, and response profiles of gut-related interoceptive EEG activity across diagnostic categories. Exploratory analyses will further examine associations between interoceptive EEG markers and clinical behavioral measures.

**Discussion:**

By systematically investigating an under assessed dimension of consciousness, this study protocol aims to establish the feasibility and signal-level characteristics of gut-related interoceptive EEG responses in patients with disorders of consciousness. By providing methodological and exploratory evidence at the group level, the findings are intended to inform the design of future hypothesis-driven and validation studies, and to support the longer-term development of complementary assessment approaches that extend beyond behavior-based evaluation, with potential relevance for clinical research and public health-oriented diagnostic strategies.

**Clinical trial registration:**

[ClinicalTrials.gov], identifier [NCT07208942].

## 1 Introduction

Disorders of consciousness (DoC), including unresponsive wakefulness syndrome (UWS) and minimally conscious state (MCS), represent devastating sequelae of severe brain injuries such as traumatic brain injury, stroke, and cardiac arrest. Advances in neurosurgery and critical care have increased survival following life-threatening brain insults; however, many patients progress to prolonged DoC, a condition associated with high mortality, severe disability, and substantial long-term care needs. As a result, DoC imposes profound physical, emotional, and economic burdens on patients’ families, healthcare systems, and society at large, contributing to a growing global population of affected individuals (1, 2). Epidemiological data indicate that approximately 4,200 new cases of UWS occur annually in the United States, while the incidence of MCS remains uncertain. Depending on diagnostic criteria, the prevalence of UWS is estimated to range from 5,000 to 42,000 individuals, whereas MCS prevalence may reach 112,000 to 280,000 individuals (3, 4). The lifetime cost of care for individuals with prolonged DoC can exceed $1,000,000 (5). Given China’s large population base, the public health burden associated with prolonged DoC is expected to be substantial. Despite this substantial burden, accurate diagnosis and clinical stratification of DoC remain among the most complex challenges in modern neurology.

The clinical classification of DoC relies predominantly on standardized behavioral assessments, with the Coma Recovery Scale–Revised (CRS-R) serving as the most widely used and validated diagnostic gold standard (6–8). The CRS-R evaluates behavioral responses to external sensory stimulation across six domains—auditory, visual, motor, oromotor, communication, and arousal—enabling differentiation between UWS, characterized by the absence of behavioral evidence of awareness, and MCS, defined by inconsistent but reproducible signs of conscious processing. Its strong psychometric properties have made it central to prognosis, treatment planning, and research enrollment (9, 10).

Nevertheless, behavior-based assessments are subject to important limitations. Their accuracy depends heavily on examiner expertise, and even trained clinicians may demonstrate inter-rater variability when scoring CRS-R items (11). More critically, cognitive–motor dissociation (CMD)—a condition in which patients retain conscious awareness but lack the motor capacity to express it—can lead to systematic underestimation of consciousness based solely on observable behavior (12). Reported misdiagnosis rates in DoC populations range from 37 to 43%, with CMD affecting approximately 8% of coma patients, 7% of UWS patients, and up to 20% of patients classified as MCS minus (MCS−) (11, 13, 14). This means one in five MCS patients may have their true level of consciousness substantially underestimated based solely on behavioral observations. Such diagnostic inaccuracies have profound ethical and clinical consequences, potentially denying patients appropriate rehabilitation opportunities or leading to unsuitable treatment decisions.

To overcome the limitations of behavioral assessments, neuroimaging techniques such as functional magnetic resonance imaging (fMRI) and fluorodeoxyglucose positron emission tomography (FDG-PET) have been increasingly employed to detect covert consciousness—neural evidence of awareness not apparent at the bedside (15–19). Although these modalities have demonstrated value in selected cases, their routine clinical use is constrained by high costs, operational complexity, and limited accessibility, particularly in resource-limited settings. Practical challenges further restrict their feasibility in severely impaired patients. For instance, fMRI requires patients to maintain immobility for prolonged periods—a nearly insurmountable barrier for DoC patients with motor deficits or agitation. Additionally, FDG-PET relies on radioactive tracers and remains largely inaccessible in low and middle-income countries (LMICs). These limitations underscore the urgent need for objective, low-cost, and portable assessment tools that can complement existing diagnostic frameworks.

From a theoretical perspective, consciousness is widely conceptualized as comprising two interacting components: arousal and awareness. Awareness itself encompasses both external awareness of the environment and internal self-awareness (20, 21). While many patients with DoC retain arousal, as evidenced by preserved sleep–wake cycles, awareness is variably impaired. Current diagnostic paradigms, however, focus almost exclusively on external awareness, assessed through sensory responsiveness and command-following tasks, while self-awareness—a core aspect of subjective experience—remains largely unexamined in routine clinical practice (22).

Interoception, defined as the brain’s capacity to sense and integrate internal bodily signals such as cardiac, respiratory, and gastrointestinal activity, is increasingly recognized as a fundamental contributor to self-awareness (23–25). Alterations in interoceptive processing have been implicated in a range of neuropsychiatric and neurological disorders (26), and emerging evidence suggests that disrupted interoceptive signaling may also play a role in altered states of consciousness (23). To date, interoceptive research in DoC has focused primarily on cardiac-related measures, such as heartbeat-evoked potentials. However, these signals can be influenced by movement artifacts, medication effects, and autonomic instability, limiting their robustness in clinical settings (27–29).

The gastrointestinal system, by contrast, represents a major interoceptive organ that communicates with the brain through the gut–brain axis, involving the vagus nerve, enteric nervous system, and multiple neurochemical pathways (30). Gut-related interoceptive signals are physiologically salient and relatively stable, yet their potential relevance to DoC assessment has not been systematically explored.

Importantly, controlled visceral stimulation paradigms, such as rectal balloon distension, have been widely used in gastrointestinal and neurophysiological research to investigate interoceptive processing (31–33). Previous studies have demonstrated that such stimulation can reliably evoke cortical responses, including activations in the insular and anterior cingulate cortices, as well as time-locked electrophysiological responses measurable with EEG and evoked potential techniques (34, 35). These findings support the feasibility of using rectal distension as a controlled and reproducible approach to probe gut-related interoceptive signaling in humans.

Electroencephalography (EEG) offers a particularly suitable platform for investigating such processes, given its low cost, portability, high temporal resolution, and feasibility for bedside application in severely impaired patients.

Against this background, the present study protocol proposes an EEG-based interoceptive assessment paradigm using controlled, non-invasive rectal balloon distension to probe gut-related interoceptive processing in patients with DoC. Given the absence of standardized interoceptive paradigms in DoC populations, a rigorously defined protocol represents a necessary first step prior to hypothesis-driven or diagnostic validation studies. The present study is designed to examine the feasibility, signal characteristics, and descriptive group-level patterns of interoceptive EEG responses across diagnostic categories. By systematically investigating an underexplored dimension of consciousness, this work aims to provide foundational, hypothesis-generating evidence that may inform future validation studies and facilitate the development of complementary assessment approaches extending beyond behavior-based diagnostics.

## 2 Methods and analysis

### 2.1 Study design and setting

This study is designed as a prospective, observational, exploratory study. Its primary objective is to evaluate the feasibility, signal characteristics, and descriptive group-level electrophysiological patterns associated with gut-related interoceptive processing in patients with DoC, rather than to establish diagnostic accuracy or individual-level classification. The study is conducted in the General Department of Hyperbaric Oxygen at the Second People’s Hospital of Hefei (Hefei, China).

**Figure 1:**
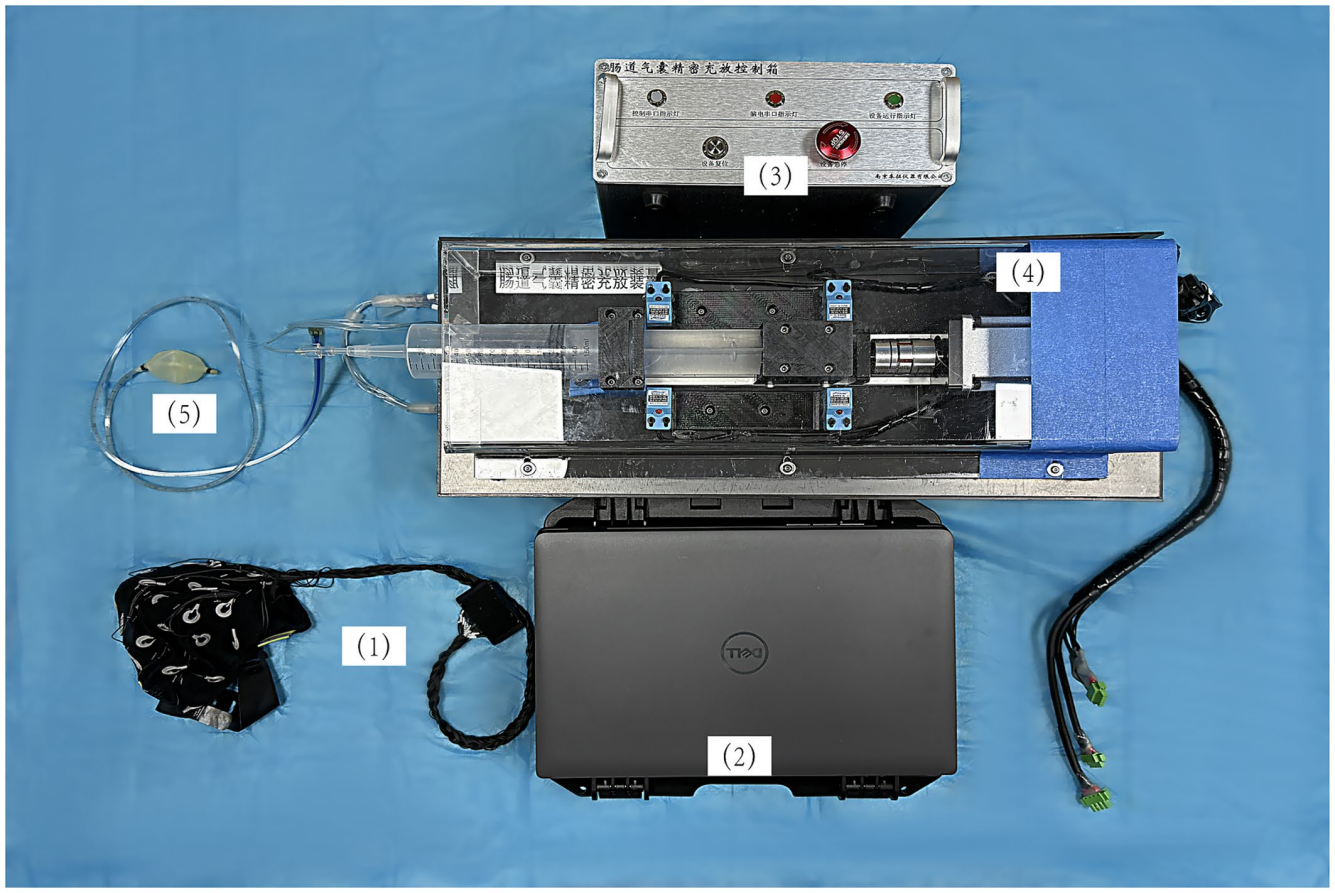
Schematic diagram of the rectal balloon stimulation and electroencephalogram synchronous recording system: (1) A 32-channel scalp electrode cap: continuous acquisition of cortical electrical signals; (2) Stimulation control and data recording computer: customized software was run to set stimulation parameters and send synchronous trigger signals, and EEG responses were recorded at the same time; (3) Intestinal airbag precision charging and discharging control device: through the servo motor; (4) Drive screw module, precise control of gas injection and withdrawal; (5) Anorectal manometry catheter: the front end was connected with an expandable balloon, lubricated with paraffin oil and placed in the rectum.

The center has established a specialized system covering diagnostic confirmation and comprehensive management, which includes standardized diagnostic workflows, a bedside electroencephalography (EEG) system (iRecorder W32), and a multidisciplinary clinical research team proficient in interoceptive testing and DoC research. Additionally, the controllable rapid inflation-deflation rectal stimulation device was independently developed by the research team (Figure 1). These resources ensure protocol adherence, data uniformity, and participant safety throughout the entire study period. The study will be reported in accordance with STARD-informed principles where applicable, with an emphasis on transparent reporting of study design, participant flow, and analytical procedures.

### 2.2 Participant enrollment

Participants will be recruited according to predefined inclusion and exclusion criteria (Table 1). The target sample size is 15 participants per group: unresponsive wakefulness syndrome (UWS), minimally conscious state (MCS), and age and sex-matched healthy controls (HCs). Written informed consent will be obtained from all participants or, for DoC patients, from their legal guardians prior to enrollment. Healthy control participants will be recruited from the local community and screened to exclude neurological, psychiatric, or gastrointestinal disorders.

**Table 1:** Inclusion and exclusion criteria for DoC and control individuals.

Patient group inclusion criteria	Patient group exclusion criteria
1. Patients with chronic-phase disorders of consciousness (DoC) (duration ≥28 days) due to etiologies such as traumatic brain injury, stroke, or cardiac arrest.	1. History of developmental psychiatric or neurological disorders causing functional impairment.
2. Age ≥18 years.	2. Untreated epilepsy.
3. Absence of acute complications such as fever or infection within 15 days prior to enrollment.	3. Untreated cerebral edema.
4. No use of sedative medications within 15 days prior to enrollment.	4. Unstable vital signs.
5. No use of any medications potentially affecting gastrointestinal motility within 7 days prior to testing.	5. History of intestinal diseases.
6. Written informed consent provided by family members or legal guardians.	6. History of abdominal surgery, except for simple appendectomy or cholecystectomy.
	7. Presence of organic diseases such as spinal cord injury or thyroid dysfunction.
	8. Patients with skull defects resulting in cranial depression, or those who have undergone cranioplasty with titanium alloy.

Control group inclusion criteria	Control group exclusion criteria
1. Age-matched, conscious healthy adults.	1. Failure to meet any of the above inclusion criteria.
2. No history of gastrointestinal diseases or abdominal surgery.	2. Unwillingness to provide written informed consent.
3. No history of psychiatric disorders.	
4. Cessation of any medications or foods affecting psychiatric or gastrointestinal function 48 h prior to the experiment.	
5. Written informed consent provided by the participant.	

Given that patients with DoC frequently present with a history of decompressive craniectomy or cranioplasty, additional criteria will be applied to ensure the quality and interpretability of EEG recordings. Patients with skull defects associated with significant cranial depression will be excluded, as such conditions may substantially distort electrophysiological signals. In addition, patients who have undergone cranioplasty using titanium implants will be excluded due to potential interference with EEG signal acquisition. In contrast, patients reconstructed with polyetheretherketone (PEEK) materials or autologous bone grafts will be considered eligible, as these materials are not expected to significantly affect EEG recording quality (36).

The study protocol has been registered on ClinicalTrials.gov (NCT07208942) and approved by the Ethics Committee of the Second People’s Hospital of Hefei (Approval No.: 2024-Research-170).

### 2.3 Study workflow

**Figure 2:**
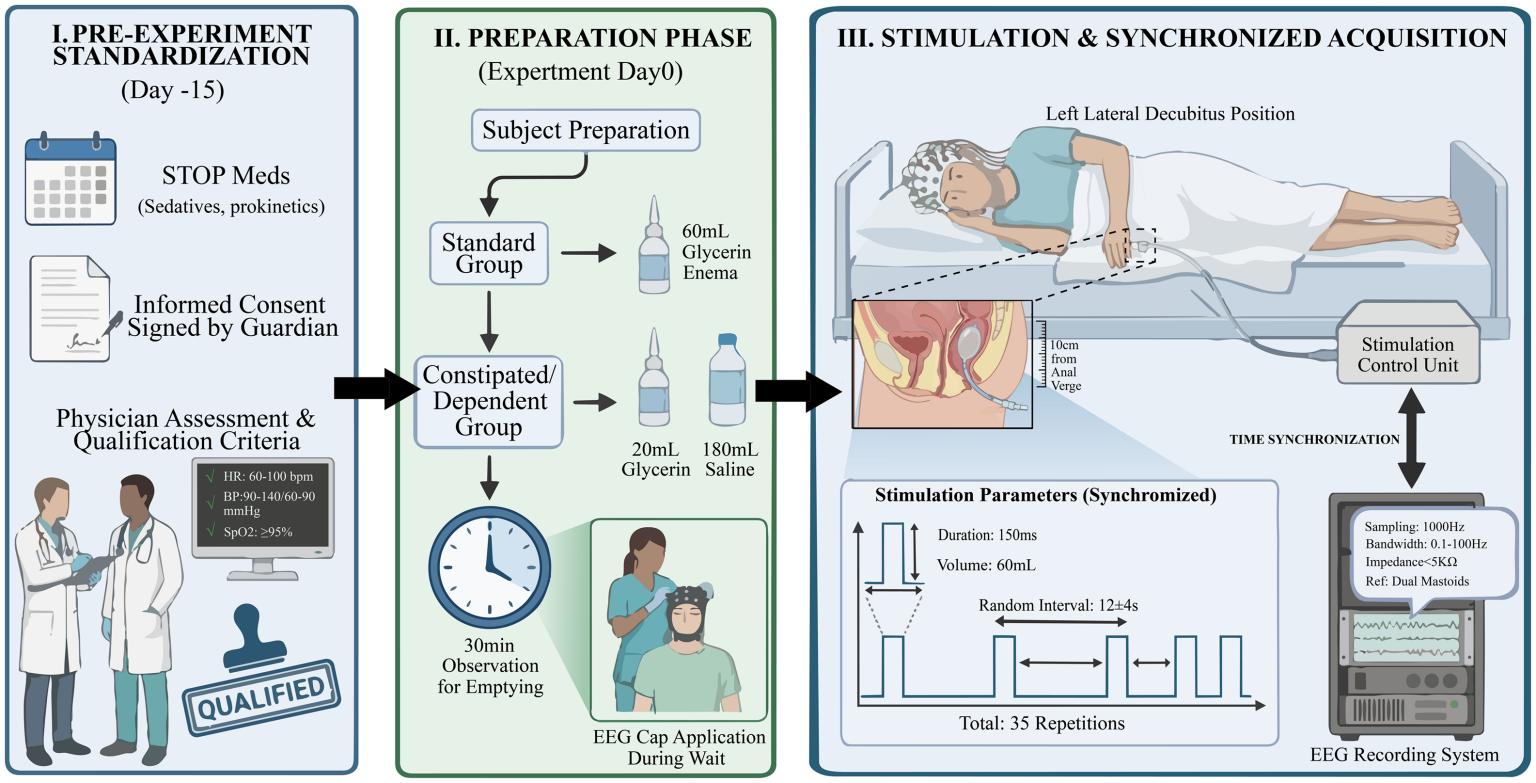
Study workflow the schematic outlines the experimental procedure for assessing EEG responses to rectal stimulation across participant groups (HC, healthy controls; MCS; UWS). Key phases include pre-experiment preparation, rectal stimulation, EEG signal acquisition, and subsequent time-frequency analysis of the event-related potentials (ERPs).

Upon enrollment (Figure 2), all DoC patients will undergo standardized CRS-R assessments administered at least five times over a one-week period by a trained multidisciplinary team. Scores across all six CRS-R subscales will be documented. Diagnostic classification will be based on the highest level of reproducible behavioral response observed during repeated assessments.

Rectal preparation, EEG setup, rectal catheter placement, stimulation parameters, EEG acquisition, and event synchronization procedures will be conducted as previously described, following standardized safety and comfort protocols. Continuous clinical monitoring will be maintained throughout the procedure. The stimulation will be immediately discontinued if signs of discomfort, autonomic instability, or adverse events are observed. All procedures will be conducted by trained clinical personnel following predefined safety and tolerability criteria, including continuous monitoring of autonomic signs (heart rate, blood pressure, oxygen saturation) and immediate termination of stimulation in case of distress or physiological instability.

### 2.4 Rectal stimulation and EEG acquisition

The experimental procedure comprises a preparatory phase and a stimulation phase. Prior to study initiation, written informed consent will be obtained from the legal guardians of all patients with disorders of consciousness (DoC). To minimize potential confounding effects on gastrointestinal motility, medications known to influence gut function (e.g., sedatives, prokinetic agents) will be discontinued for at least 15 days before the experiment, where clinically feasible and under medical supervision.

Before participation, all patients will be jointly evaluated by attending neurologists and gastroenterologists to ensure clinical stability. Inclusion requires stable vital signs, defined as a heart rate of 60–100 beats/min, blood pressure of 90–140/60–90 mmHg, and oxygen saturation of ≥95%.

#### 2.4.1 Bowel preparation and setup

Prior to stimulation, rectal preparation will be performed using standardized protocols. A 60 mL glycerin enema will be administered in the standard condition. For participants with chronic constipation or long-term enema dependence, a combined preparation (20 mL glycerin + 180 mL normal saline) will be used. Following bowel evacuation, a 30-min observation period will be implemented to ensure rectal clearance and physiological stabilization. During this period, the EEG cap will be positioned and impedance optimized.

#### 2.4.2 Rectal stimulation paradigm

Gut-related interoceptive stimulation will be delivered using a controlled rectal balloon distension paradigm. A lubricated balloon-tipped catheter (liquid paraffin) will be inserted such that the distal balloon is positioned approximately 10 cm from the anal verge, guided by catheter scale markings. Participants will be maintained in a left lateral decubitus position throughout the procedure.

Stimulation will be administered using a computer-controlled inflation–deflation device developed with participation from the research team, ensuring precise temporal and volumetric control. Each trial will consist of rapid balloon inflation to a fixed volume of 60 mL, reaching target volume within 150 ms, followed by passive deflation.

The inter-stimulus interval (ISI) will be pseudo-randomized at 12 ± 4 s to minimize anticipatory responses and neural habituation. A total of 35 stimulation trials will be delivered per participant, resulting in an approximate stimulation duration of 7–9 min.

#### 2.4.3 EEG acquisition parameters

Continuous EEG will be recorded using a 32-channel system (iRecorder W32, Niantong Intelligent Technology, Shanghai, China). Electrodes will be positioned according to the international 10–20 system, with particular emphasis on fronto-central and parietal regions associated with interoceptive and cognitive processing.

A wet electrode cap based on Ag/AgCl cup electrodes will be used. Conductive gel will be applied to each electrode site to reduce scalp impedance, which will be maintained below 5 kΩ throughout the recording. EEG signals will be sampled at 1000 Hz, with an online bandwidth of 0.1–100 Hz. Signals will be referenced to the bilateral mastoids, with the ground electrode placed at a standard frontal location.

Strict data quality control procedures will be implemented throughout acquisition, including continuous impedance monitoring and artifact inspection.

#### 2.4.4 Event synchronization

To ensure temporal alignment between physiological stimulation and neural responses, event synchronization will be implemented via serial communication-based triggering. Specifically, the rectal stimulation device will transmit event markers through a serial interface to the EEG acquisition system at the onset of each balloon inflation.

These event markers will be embedded in real time within the EEG data stream, enabling time-locking of electrophysiological signals to stimulation events for subsequent event-related potential (ERP) and time–frequency analyses.

These trigger signals will be embedded as event markers within the EEG data stream, enabling millisecond-level temporal alignment for subsequent event-related potential (ERP) and time–frequency analyses. This hardware-based synchronization approach ensures high temporal precision and reproducibility of stimulus–response coupling.

All procedures will be conducted under continuous clinical monitoring. Stimulation will be immediately discontinued if any signs of discomfort, autonomic instability, or adverse physiological responses are observed.

### 2.5 Sample size estimation

An *a priori* power analysis was conducted using G*Power (v3.1.9.7) based on pilot ERP amplitude data (*n* = 4 per group). Using an independent-samples t-test model, the pilot data yielded a Cohen’s d of 1.13. With a two-tailed *α* = 0.05 and 80% power, the minimum required sample size was estimated at 13 participants per group. To account for potential data loss or dropout, the target sample size was increased to 15 participants per group.

Given the exploratory nature of this study, the sample size is intended to support feasibility assessment and preliminary effect size estimation rather than definitive diagnostic accuracy evaluation. The pilot-based power calculation was performed using a pairwise comparison framework to estimate effect size magnitude; group-level comparisons in the present study are intended for descriptive and exploratory purposes rather than confirmatory hypothesis testing.

### 2.6 Data preprocessing and statistical analysis

EEG data will be preprocessed using EEGLAB (v2024.0) in MATLAB R2024b following a predefined pipeline. Continuous data will be band-pass filtered between 0.1 and 40 Hz and segmented into epochs time-locked to rectal distension onset (−200 to 800 ms). Independent component analysis (ICA) will be applied to identify and remove components associated with ocular, muscular, and cardiac artifacts, based on spatial topography, power spectra, and time-course characteristics.

Event-related potentials (ERPs) will be quantified at predefined fronto-central electrodes. ERP components of interest (N1, P2, and P3) will be defined within fixed latency windows based on prior interoceptive ERP literature (N1: 80–150 ms; P2: 150–250 ms; P3: 250–400 ms). Mean amplitude and peak latency within these windows will be extracted for statistical analysis.

Time–frequency analyses will be conducted using event-related spectral perturbation (ERSP) methods, with baseline normalization relative to the −200 to 0 ms pre-stimulus interval. Power changes in the theta (4–7 Hz) and alpha (8–12 Hz) bands will be quantified.

Group-level comparisons among UWS, MCS, and healthy control participants will be performed using one-way ANOVA for each predefined electrophysiological outcome. *Post hoc* pairwise comparisons will be corrected for multiple testing using the Tukey HSD procedure. Effect sizes (η^2^ or Cohen’s d, as appropriate) will be reported to inform the design of future confirmatory studies. All analyses will be restricted to predefined electrodes, frequency bands, and latency windows to limit multiple comparisons and reduce the risk of false-positive findings.

### 2.7 Aims and outcome measures

The primary aim of this study is to assess the feasibility of recording gut-related interoceptive EEG responses in patients with disorders of consciousness under standardized stimulation conditions.

The primary outcomes are predefined electrophysiological measures, including:

(1) ERP component amplitude and latency (N1, P2, P3) time-locked to rectal distension, and.
(2) Event-related spectral perturbation (ERSP) in the theta (4–7 Hz) and alpha (8–12 Hz) frequency bands.

Secondary exploratory outcomes include group-level comparisons of these electrophysiological measures among UWS, MCS, and healthy control participants, as well as exploratory associations between EEG markers and CRS-R total scores and subscale scores.

All outcomes are exploratory and group-level in nature. This study is not designed to support individual-level diagnostic classification or clinical decision-making, but rather to assess feasibility and to provide preliminary effect size estimates that may inform the design of future hypothesis-driven and validation studies.

### 2.8 Patient and public involvement

Patients and members of the public were not involved in the design, conduct, reporting, or dissemination of this study.

### 2.9 Ethics

This study protocol has been registered on ClinicalTrials.gov (NCT07208942) and approved by the Ethics Committee of the Second People’s Hospital of Hefei (Approval No: 2024-Research-170). The study will be conducted in strict adherence to the Declaration of Helsinki and local regulatory requirements to ensure participant safety and ethical integrity.

Prior to initiating any study procedures, written informed consent will be obtained from all participants (or their legal guardians, for patients with DoC). To ensure comprehension, consent information will be provided in both written (simplified Chinese) and oral formats, with opportunities for questions to be addressed by the study team. For DoC patients unable to provide consent, approval will be sought from an immediate family member or legal representative—who will receive a detailed explanation of the study’s purpose, procedures, risks and benefits.

To protect participant privacy, all data (case report forms, CRS-R assessments, EEG recordings, rectal manometry results) will be labeled only with unique participant codes (no names, medical record numbers, or other). Physical documents will be stored in locked filing cabinets in a restricted-access room; electronic data will be saved on password-protected, study-specific computers with access limited to authorized personnel (co-investigators, data managers). Personally identifiable information will not be shared with third parties, including commercial entities or non-study clinicians. Research-related personal data will be retained for 5 years following the publication of final results and then securely destroyed in compliance with institutional policies and China’s Personal Information Protection Law.

## 3 Discussion

The present study protocol addresses a persistent and clinically consequential limitation in current approaches to the assessment of disorders of consciousness (DoC): the lack of objective methods capable of probing dimensions of consciousness that extend beyond overt behavioral responsiveness. Despite substantial advances in neurocritical care, routine diagnostic practice continues to rely predominantly on behavior-based assessments, which are inherently vulnerable to motor impairment, fluctuating arousal, and cognitive–motor dissociation. These limitations have been widely recognized and have important implications for diagnostic classification, prognostication, and care planning.

From a theoretical standpoint, contemporary models of consciousness emphasize that conscious experience is not defined solely by externally directed awareness, but also by internal self-related processes, including interoceptive perception. Interoception represents a core neurobiological substrate of self-awareness and is supported by distributed neural systems involving the insular cortex, anterior cingulate cortex, and brainstem arousal networks. However, this dimension remains largely absent from existing diagnostic paradigms for DoC. By proposing an EEG-based gut-related interoceptive assessment, the present protocol aims to explore the feasibility of capturing neural responses to internal bodily signals in DoC patients, thereby providing a complementary perspective to externally driven evaluation frameworks.

Methodologically, this study is explicitly designed as an exploratory and feasibility-focused investigation rather than a confirmatory diagnostic accuracy trial. The use of standardized rectal balloon distension combined with synchronized EEG acquisition enables controlled activation of the gut–brain axis while remaining compatible with bedside clinical settings. Compared with cardiac-based interoceptive measures, gut-related interoceptive signals may be less susceptible to transient autonomic fluctuations and external noise, which could represent a potential methodological advantage in severely brain-injured populations. Importantly, the present protocol prioritizes signal characterization, group-level pattern description, and preliminary effect size estimation, with the primary goal of informing the design of future hypothesis-driven and validation studies.

From a public health perspective, the potential relevance of this work extends beyond individual-level assessment. Advanced neuroimaging techniques such as fMRI and FDG-PET, while informative, are costly, resource-intensive, and often inaccessible in low and middle-income settings. EEG-based paradigms that can be implemented at the bedside offer a more scalable and equitable approach to DoC research. If feasibility and systematically characterizable neural responses are demonstrated, this line of research may contribute to the development of accessible assessment strategies aligned with public health priorities of feasibility, equity, and responsible resource utilization.

Several limitations of the present protocol should be acknowledged. The relatively small sample size and single-center design limit generalizability and preclude conclusions regarding diagnostic performance or individual-level classification. Moreover, interoceptive processing may be influenced by factors such as medication exposure, autonomic dysfunction, and gastrointestinal comorbidities, which will require systematic control in future studies. In addition, this protocol is not designed to establish causal relationships between interoceptive neural responses and conscious awareness. These considerations underscore the exploratory nature of the present work and highlight the need for subsequent multicenter and longitudinal investigations with larger cohorts and predefined validation frameworks.

Importantly, this protocol is intended to serve as an initial methodological step within a staged translational research pathway rather than as a diagnostic validation study. The primary objective is to establish feasibility, tolerability, and signal-level characteristics of gut-related interoceptive EEG responses under standardized bedside conditions, thereby generating essential methodological parameters and effect size estimates to guide subsequent multicenter and hypothesis-driven investigations.

### 3.1 Prospects for scalable and equitable diagnostics

Building on the outcomes of this protocol, future investigations are expected to progress toward multicenter studies with larger and more heterogeneous cohorts, in which interoceptive EEG markers can be systematically evaluated for their added value in patient stratification and diagnostic enrichment. Rather than serving as a standalone diagnostic tool, gut-related interoceptive EEG paradigms may ultimately function as a complementary modality alongside behavioral assessment and existing neurophysiological measures, particularly for probing neural responses potentially related to internal aspects of awareness in patients with limited or absent motor output.

From a public health perspective, the long-term translational relevance of this approach lies in its potential scalability and accessibility. As a low-cost, portable, and bedside-compatible technique, EEG-based interoceptive assessment may be particularly well suited for implementation in resource-limited settings, where advanced neuroimaging modalities are often unavailable. If validated in future studies, such paradigms could contribute to more equitable and standardized evaluation pathways for disorders of consciousness, supporting responsible allocation of rehabilitative resources and improving population-level care strategies.

### 3.2 Potential risks, burdens, and benefits

The proposed protocol involves controlled rectal balloon distension combined with EEG recording, which warrants careful consideration of potential risks, participant burden, and anticipated benefits. Rectal distension may induce transient discomfort or mild autonomic responses, such as changes in heart rate or blood pressure. However, the stimulation parameters employed are comparable to procedures routinely used in clinical bowel care for patients with prolonged disorders of consciousness. All procedures will be conducted under continuous bedside monitoring by trained clinical personnel, with predefined stopping criteria and immediate access to medical support to ensure participant safety.

Overall participant burden is considered low. EEG recording is non-invasive and widely used in routine neurological practice, and the rectal stimulation protocol is time-limited and integrated into standard nursing workflows. Importantly, participation in this study will not alter routine clinical management or influence diagnostic or therapeutic decision-making for enrolled patients.

While no direct clinical benefit to individual participants can be expected, the potential scientific and societal benefits are meaningful. By systematically exploring an underexamined dimension of consciousness, this study may provide foundational evidence to inform future research aimed at developing more comprehensive, accessible, and theoretically grounded assessment approaches for DoC. In the longer term and at the population level, such advances may support improved research stratification, more informed prognostic discussions, and the responsible allocation of healthcare resources, in line with public health priorities.
